# Supplementary material for: Multi‐dataset identification of innovative feature genes and molecular mechanisms in keratoconus
Source: J Cell Mol Med. 2024 Sep 19;28(18):e70079. doi: 10.1111/jcmm.70079 (PMC11412914; doi:10.1111/jcmm.70079)
Supplement: Supplementary file 2 — Material S2. [file JCMM-28-e70079-s002.zip › 7.GSEA analysis/4.MYRF/KEGG_DILATED_CARDIOMYOPATHY.html]

Details for gene set KEGG\_DILATED\_CARDIOMYOPATHY[GSEA]

|  || Dataset | MYRF.group.cls#h\_versus\_l |
| Phenotype | group.cls#h\_versus\_l |
| Upregulated in class | l |
| GeneSet | KEGG\_DILATED\_CARDIOMYOPATHY |
| Enrichment Score (ES) | -0.37875164 |
| Normalized Enrichment Score (NES) | -1.3877152 |
| Nominal p-value | 0.0392562 |
| FDR q-value | 0.25513166 |
| FWER p-Value | 0.894 |
Table: GSEA Results Summary

  

Fig 1: Enrichment plot: KEGG\_DILATED\_CARDIOMYOPATHY      
 Profile of the Running ES Score & Positions of GeneSet Members on the Rank Ordered List

  

| SYMBOL | TITLE | RANK IN GENE LIST | RANK METRIC SCORE | RUNNING ES | CORE ENRICHMENT || 1 | TNNT2 | na | 75 | 0.896 | 0.0462 | No |
| 2 | CACNG8 | na | 755 | 0.506 | 0.0539 | No |
| 3 | TNNI3 | na | 1269 | 0.424 | 0.0620 | No |
| 4 | CACNG5 | na | 1534 | 0.398 | 0.0758 | No |
| 5 | ITGA4 | na | 1618 | 0.389 | 0.0944 | No |
| 6 | ADCY1 | na | 1675 | 0.385 | 0.1136 | No |
| 7 | PLN | na | 2393 | 0.329 | 0.1106 | No |
| 8 | TTN | na | 2608 | 0.317 | 0.1216 | No |
| 9 | ADCY2 | na | 2688 | 0.312 | 0.1362 | No |
| 10 | ACTC1 | na | 3638 | 0.266 | 0.1232 | No |
| 11 | ADCY5 | na | 4506 | 0.238 | 0.1110 | No |
| 12 | DAG1 | na | 4906 | 0.225 | 0.1116 | No |
| 13 | MYBPC3 | na | 5513 | 0.207 | 0.1053 | No |
| 14 | CACNB1 | na | 5538 | 0.206 | 0.1157 | No |
| 15 | ITGA9 | na | 5848 | 0.197 | 0.1174 | No |
| 16 | TNNC1 | na | 8243 | 0.132 | 0.0555 | No |
| 17 | ADCY7 | na | 8605 | 0.125 | 0.0518 | No |
| 18 | IGF1 | na | 8743 | 0.121 | 0.0544 | No |
| 19 | EMD | na | 9064 | 0.115 | 0.0513 | No |
| 20 | ITGB7 | na | 9321 | 0.109 | 0.0498 | No |
| 21 | ADCY4 | na | 9919 | 0.097 | 0.0379 | No |
| 22 | TPM4 | na | 10259 | 0.091 | 0.0330 | No |
| 23 | CACNB2 | na | 10834 | 0.081 | 0.0208 | No |
| 24 | MYH6 | na | 11121 | 0.076 | 0.0166 | No |
| 25 | CACNA2D3 | na | 11412 | 0.072 | 0.0121 | No |
| 26 | DES | na | 11440 | 0.071 | 0.0152 | No |
| 27 | PRKX | na | 11663 | 0.068 | 0.0125 | No |
| 28 | CACNA1C | na | 11956 | 0.064 | 0.0075 | No |
| 29 | TPM1 | na | 12030 | 0.063 | 0.0088 | No |
| 30 | ITGB6 | na | 12239 | 0.060 | 0.0061 | No |
| 31 | ACTB | na | 12485 | 0.057 | 0.0020 | No |
| 32 | MYH7 | na | 12644 | 0.055 | 0.0004 | No |
| 33 | ADCY6 | na | 13405 | 0.045 | -0.0191 | No |
| 34 | CACNA1D | na | 13802 | 0.040 | -0.0283 | No |
| 35 | ATP2A2 | na | 14195 | 0.036 | -0.0377 | No |
| 36 | DMD | na | 14406 | 0.033 | -0.0420 | No |
| 37 | CACNG7 | na | 16278 | 0.015 | -0.0952 | No |
| 38 | TPM2 | na | 17462 | 0.004 | -0.1291 | No |
| 39 | ADCY9 | na | 17667 | 0.003 | -0.1349 | No |
| 40 | CACNG1 | na | 17979 | -0.000 | -0.1438 | No |
| 41 | CACNG2 | na | 18631 | -0.007 | -0.1623 | No |
| 42 | ITGB4 | na | 19208 | -0.013 | -0.1782 | No |
| 43 | PRKACA | na | 19584 | -0.017 | -0.1881 | No |
| 44 | ADCY8 | na | 20359 | -0.026 | -0.2091 | No |
| 45 | LMNA | na | 20403 | -0.027 | -0.2089 | No |
| 46 | TPM3 | na | 20434 | -0.027 | -0.2083 | No |
| 47 | MYL3 | na | 20790 | -0.031 | -0.2169 | No |
| 48 | ADRB1 | na | 22106 | -0.047 | -0.2523 | No |
| 49 | TGFB2 | na | 22177 | -0.048 | -0.2517 | No |
| 50 | ACTG1 | na | 22317 | -0.050 | -0.2531 | No |
| 51 | GNAS | na | 22466 | -0.052 | -0.2545 | No |
| 52 | LAMA2 | na | 23159 | -0.062 | -0.2712 | No |
| 53 | ITGA3 | na | 23712 | -0.071 | -0.2833 | No |
| 54 | ITGB5 | na | 23730 | -0.071 | -0.2799 | No |
| 55 | ITGA2 | na | 25150 | -0.096 | -0.3157 | No |
| 56 | ITGA2B | na | 25913 | -0.111 | -0.3317 | No |
| 57 | CACNB3 | na | 26225 | -0.118 | -0.3343 | No |
| 58 | ITGAV | na | 26862 | -0.132 | -0.3456 | No |
| 59 | ADCY3 | na | 26955 | -0.134 | -0.3410 | No |
| 60 | SGCA | na | 27650 | -0.152 | -0.3529 | No |
| 61 | ITGA11 | na | 28180 | -0.166 | -0.3592 | No |
| 62 | SGCG | na | 28859 | -0.187 | -0.3687 | Yes |
| 63 | CACNA1F | na | 28862 | -0.187 | -0.3587 | Yes |
| 64 | SGCD | na | 29214 | -0.197 | -0.3581 | Yes |
| 65 | ITGA7 | na | 29295 | -0.200 | -0.3497 | Yes |
| 66 | ITGA8 | na | 29346 | -0.201 | -0.3403 | Yes |
| 67 | ITGA6 | na | 29414 | -0.203 | -0.3312 | Yes |
| 68 | CACNG6 | na | 29465 | -0.205 | -0.3216 | Yes |
| 69 | ITGB3 | na | 29529 | -0.207 | -0.3123 | Yes |
| 70 | ITGB1 | na | 29873 | -0.219 | -0.3103 | Yes |
| 71 | RYR2 | na | 30209 | -0.231 | -0.3075 | Yes |
| 72 | MYL2 | na | 30601 | -0.246 | -0.3056 | Yes |
| 73 | ITGB8 | na | 30931 | -0.260 | -0.3010 | Yes |
| 74 | PRKACG | na | 31385 | -0.285 | -0.2987 | Yes |
| 75 | CACNG4 | na | 31907 | -0.312 | -0.2970 | Yes |
| 76 | CACNA2D2 | na | 32005 | -0.317 | -0.2826 | Yes |
| 77 | ITGA10 | na | 32096 | -0.323 | -0.2678 | Yes |
| 78 | SGCB | na | 32475 | -0.349 | -0.2599 | Yes |
| 79 | CACNA2D4 | na | 32488 | -0.350 | -0.2413 | Yes |
| 80 | TGFB1 | na | 32868 | -0.377 | -0.2319 | Yes |
| 81 | TGFB3 | na | 33024 | -0.389 | -0.2154 | Yes |
| 82 | CACNA1S | na | 33196 | -0.406 | -0.1984 | Yes |
| 83 | SLC8A1 | na | 33227 | -0.410 | -0.1771 | Yes |
| 84 | PRKACB | na | 33438 | -0.435 | -0.1597 | Yes |
| 85 | ITGA1 | na | 33768 | -0.482 | -0.1432 | Yes |
| 86 | CACNB4 | na | 34188 | -0.569 | -0.1246 | Yes |
| 87 | CACNG3 | na | 34232 | -0.580 | -0.0945 | Yes |
| 88 | TNF | na | 34328 | -0.609 | -0.0644 | Yes |
| 89 | ITGA5 | na | 34505 | -0.679 | -0.0328 | Yes |
| 90 | CACNA2D1 | na | 34582 | -0.726 | 0.0042 | Yes |
Table: GSEA details [plain text format]

  

Fig 2: KEGG\_DILATED\_CARDIOMYOPATHY      
 Blue-Pink O' Gram in the Space of the Analyzed GeneSet

  

Fig 3: KEGG\_DILATED\_CARDIOMYOPATHY: Random ES distribution      
 Gene set null distribution of ES for **KEGG\_DILATED\_CARDIOMYOPATHY**
